# Paeoniflorin regulates the hypothalamic-pituitary-adrenal axis negative feedback in a rat model of post-traumatic stress disorder

**DOI:** 10.22038/ijbms.2020.41214.9738

**Published:** 2020-04

**Authors:** Jie Chen, Weiqiong Ye, Ling Li, Junfang Su, Yunling Huang, Lingyun Liu, Lili Wu, Can Yan

**Affiliations:** 1 The Research Centre of Basic Integrative Medicine, Guangzhou University of Chinese Medicine, Guangzhou 510006, China; 2 Department of Pathology and Pathophysiology, School of Basic Medical Sciences, Guangzhou University of Chinese Medicine, Guangzhou, China

**Keywords:** Adrenocorticotropin – hormone, Behavior, Corticosterone, Corticotropin-releasing – hormone, Hypothalamic-pituitary-adrenal axis, Paeoniflorin, Post-traumatic stress - disorder

## Abstract

**Objective(s):**

To investigate the effects of paeoniflorin (PEF) on the hypothalamic-pituitary-adrenal (HPA) axis feedback function of post-traumatic stress disorder (PTSD).

**Materials and Methods:**

Single-prolonged stress (SPS) was used to establish a PTSD-like rat model. The contents of plasma corticosterone (CORT), adrenocorticotropin hormone (ACTH) and corticotropin-releasing hormone (CRH) were measured by ELISA. Glucocorticoid receptor (GR), mineralocorticoid receptor (MR), adrenocorticotropic hormone-releasing factor I receptor (CRF1R), and adrenocorticotropic hormone-releasing factor II receptor (CRF2R) in the hippocampus and amygdala were measured by RT-PCR and immunohistochemistry.

**Results:**

The results showed that on day 8 after SPS, model rats showed enhanced HPA axis negative feedback lasting to day 29. On day 29, plasma CORT levels increased in model rats, while plasma CRH levels had no significant difference on days 8, 22, and 29. The expression of GR and MR of model rats significantly increased in the hippocampus, while the expression of GR, MR, and CRF1R significantly decreased in the amygdala. After 14 days of continuous administration of PEF, the enhanced negative feedback was inhibited, and the plasma CORT level significantly reduced after 21 days of administration. Moreover, PEF could significantly decrease the expression of GR and MR in the hippocampus, and increase the expression of GR, MR, and CRF1R significantly in the amygdala.

**Conclusion:**

PEF could regulate HPA axis dysfunction in a rat model of PTSD, which may be related to regulating expression of GR and MR in the hippocampus and amygdala and regulating expression of CRF1R in the amygdala.

## Introduction

Post-traumatic stress disorder (PTSD) is a delayed serious physical and mental disorder following exposure to a severe traumatic stressor. Core symptoms include persistent experiencing of trauma, hypervigilance, and avoidance. Beyond that, anxiety, depression, and fear are common clinical symptoms (1). PTSD has become a focus in society for its increasing morbidity, difficultly in treatment, and high lifetime prevalence (2, 3). Currently, antidepressants are mainly used in PTSD therapy, including selective serotonin reuptake inhibitors (SSRIs) and serotonin and norepinephrine reuptake inhibitors (SNRIs) such as paroxetine (PRX) and venlafaxine (4). But these antidepressants cannot cure PTSD completely. Some symptoms persist after long-term treatment. In addition, sustained use of such drugs could have significant side effects (5). Therefore, it is necessary to find safe and effective anti-PTSD drugs. Corticosterone (CORT) is an indicator of the function of the hypothalamic-pituitary-adrenal (HPA) axis. Usually, stress exposure triggers an emotional response that activates the hypothalamus through connection to the limbic system and then triggers the activity of the HPA axis. Specific neurons in the parvocellular paraventricular nucleus (PVN) produce the corticotropin-releasing hormone (CRH) which subsequently stimulates release of adrenocorticotropin hormone (ACTH) from the pituitary. ACTH is transported to the adrenal cortex via blood circulation to promote the release of cortisol. The processes of elevated cortisol levels inhibit the function of the pituitary, hypothalamus, and hippocampus through a negative feedback mechanism known as the HPA axis negative feedback function (6). It indicates that the HPA axis plays an important role in stress response and maintenance of homeostasis. However, both clinical and rodent studies of PTSD have different CORT results; CORT levels in peripheral blood are high or low or unchanged (7-11). Since PTSD is a delayed serious physical and mental disorder, different detecting time may affect the results of CORT levels. Despite different CORT levels, enhanced negative feedback of the HPA axis is an admitted pathological basis for PTSD (12). Therefore, regulating the HPA axis dysfunction plays an important role in treatment of PTSD. 

Paeoniflorin (PEF, molecular formula: C_23_H_28_O_11_), extracted from *Radix Paeoniae Alba*, belongs to monoterpenoid glycosides. It has been reported that PEF can alleviate anxiety and depressive-like behaviors, regulate the function of monoamine neurotransmitters, and protect nerves and has the advantage of low toxicity (13-15). However, to our knowledge, the effect of PEF on HPA axis negative feedback has not been reported so far. Single-prolonged stress (SPS) is a widely used paradigm to establish the PTSD model (16). SPS-induced rat model simulates PTSD-like symptoms such as anxiety, depression, irritability, hyperarousal, and lack of learning ability and memory (17). The hippocampus and amygdala are the high regulation centers of the HPA axis, which plays a major role in the regulation of basic and the HPA axis negative feedback (18, 19). This study would investigate the changes of behaviors, the HPA axis function and part of its regulating mechanisms, as well as the effects of PEF, which may reveal the potential value of PEF in treating PTSD.

## Materials and Methods

***Animals and housing***

All 355 SPF grade male Sprague-Dawley rats (180–220 g) were purchased from the animal center of Southern Medical University. Rats were group-housed (five per cage) in a strictly controlled room (23±2 ^°^C, 55±5% humidity, and 12-hr light/12-hr dark cycle), and with *ad libitum* access to food and water during the experiments. Rats were randomly divided into four groups: control group (Control), SPS group (SPS), SPS+ paeoniflorin group (PEF), and SPS+ paroxetine group (PRX). All animal experiments were performed in accordance with the requirements of the Animal Experimentation Ethics Committee of Guangzhou University of Chinese Medicine.

***Preparation and administration of drugs***

PEF (40 mg/kg; Dilger co., Nanjing, China, Lot: D17031002) and PRX (10 mg/kg; Huahai Pharmaceutical Co., LTD., Zhejiang, China, Lot: H20031106) were dissolved in distilled water and administered orally in a volume of 10 ml/kg for 14 or 21 days. The control group and SPS group were given the same amount of distilled water for 14 or 21 days. Dexamethasone phosphate injection (3 mg/kg, DEX; Suicheng pharmaceutical co., Xinzheng, China, Lot: H41021255) were dissolved in 5% glucose (Guandong kelun pharmaceutical co., Meizhou, China, Lot: H51020636) before use. 

***Preparation of the PTSD model in rats***

The rat model of PTSD was established by SPS, the most widely used method (20). The procedure was as follows: after 7 days of adaptive phase, except for the control group, all rats were restrained for 2 hr with an organic glass rigid bound device (diameter: 6 cm, length: 20 cm). Following immobilization, rats were immediately subjected to forced swim for 20 min in an organic glass cylinder (50 cm height, 20 cm diameter) filled to 35 cm height with freshwater (24±1 ^°^C), after a 15 min rest, the rats were anesthetized with ether to a deep coma (namely consciousness lost). Finally, rats were enclosed in their original cages, without disturbance, to revive. All rats were left undisturbed for 7 days and control group rats remained in their home cages. 

***Behavioral tests***

**Figure 1:**
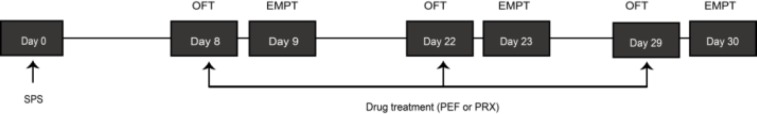
Behavioral test schedules. Rats were subjected to SPS on day 0. After 7 days of undisturbed period, paeoniflorin (PEF) or paroxetine (PRX) was administered daily from day 8 to day 22 or day 8 to day 29. Rats performed testing sessions including open field test (OFT) and elevated plus maze test (EPMT)

On days 8, 22, and 29, after SPS, behavioral tests including open field test (OFT) and elevated plus maze test (EPMT) were conducted between 5:00 pm and 11:00 pm. EPMT was carried out 24 hr after OFT. The behavioral test schedules are shown in Figure 1.

*Open field test (OFT)*

OFT is a classic animal test to evaluate anxiety-like behavior and locomotor activity. Briefly, the OFT apparatus was provided by Feidi biotechnology co. (Guangzhou, China; Lot: TM-ZFT) and consisted of a plastic box (100 cm×100 cm×40 cm), a digital camera, and computer software. Rats were placed in the center of the box and total distance in 5 min would be given to reflect the locomotor activity. In order to reduce the disturbance of animal odors, open field arenas were wiped with 1% benzalkonium bromide solution and 75% ethanol between experiments. 

*Elevated plus-maze test (EPMT)*

EPMT is used to estimate anxiety-like behavior in rats. The equipment was 36 cm off the ground. It consisted of two crisscross open arms (15 cm×48 cm) and closed arms (15 cm×48 cm×40 cm) and a central platform (15 cm×15 cm). A camera over the arena was used to record the behaviors in 5 min. The equipment was provided by Feidi biotechnology co. (Guangzhou, China; Lot: TM-GJT). Reduction in the percentage of entries, distance, and time in open arms were considered to be associated with higher levels of anxiety (21). At the beginning of each trial, the arenas were cleaned by a 1% benzalkonium bromide solution and 75% ethanol and then the subjects were positioned in the center platform facing an open arm.

***Dexamethasone suppression test (DST)***

**Figure 2:**
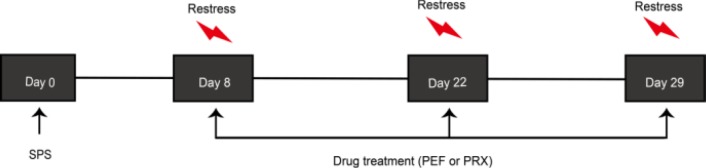
Dexamethasone suppression test (DST) schedules. Rats were subjected to SPS on day 0. After 7 days of undisturbed period, PEF or PRX was administered daily from day 8 to 22 or day 8 to 29. On days 8, 22, and 29, all rats were subjected to restress restraining, 30 min after subcutaneously injected with DEX. PRX: paroxetine; SPS: single prolonged stress; PEF: paeoniflorin: DEX: dexamethasone

Dexamethasone suppression test (DST) is a stress-restress paradigm to test plasma ACTH levels, which reflect HPA axis negative feedback. As shown in Figure 2, DST was conducted on days 8 and 22 after SPS during 8:00 am–10:00 am as follows: all rats were subjected to DEX injection subcutaneously, and then restrained 30 min (namely restress) with an organic glass rigid bound device used for SPS before. After restress, infraorbital vein blood was collected into a 10 ml EDTA tube and centrifuged at 3000 rpm for 10 min. The supernatant was collected into another 1 ml new tube and stored at -80 ^°^C refrigerator. ACTH ELISA kit (Cusabio Biotech Co., Wuhan, China, Lot: CSB-EO6875r) was used to detect plasma ACTH contents, and experimental procedures completely complied with ELISA instructions. 

***CORT and CRH detection***

Plasma CORT and CRH levels indicate the basic function of the HPA axis. Elevated CORT and CRH levels are associated with hyperfunction of the HPA axis (22). In brief, after a mild anesthesia with ether, infraorbital vein blood was collected as described above on days 8, 22, and 29 after SPS from 8:00 am–10:00 am. Corticosterone ELISA kit (Abcam, USA, Lot: ab108821) and corticotropin-releasing hormone (CRH) ELISA kit (CUSABIO, China, Lot: CSB-E08038r) were used to detect CORT and CRH levels. Experimental procedures were in accordance with ELISA instructions. In order to avoid the interaction between experiments, behavioral tests including OFT and EPMT, CORT detection, and DST were performed separately. Moreover, the animals used at each time point were in separate batches.

***Preparation of brain tissue and RT-PCR for detection of GR mRNA, MR mRNA, CRF1R mRNA, and CRF2R mRNA***

29 days after SPS, the rats were decapitated after anesthesia with chloral hydrate, the whole brain was removed rapidly and dissected on ice, afterward, the right hippocampus was stripped and the right amygdala was removed. The samples were put in an EP tube containing triazole already and then saved in -80 ^°^C refrigerator. The locations of the hippocampus and amygdala were conducted according to the rat brain in stereotaxic coordinates (23). The hippocampus was located 2.96–6.70 mm far from the ear line, the amygdala was located 5.70–7.20 mm and 4.0~5.5 mm far from the ear line and the midline, respectively.

Total RNAs were extracted from 50–100 mg brain tissue by Trizol, and reverse transcription and amplification were performed according to TAKARA instruction (PrimeScript™ RT reagent kit, Japan, Lot: RR037A; TB Green™ Premix Ex Taq™ II, Japan, Lot: RR820A). Primers for PCR amplification were designed and synthesized by Rubio Biotechnology co., LTD, in Guangzhou, the primer sequences used were as follows: β-actin, 5’-TCA AGA TCA TTG CTC CTC CTG AG-3’ (sense), 5’-ACA TCT GCT GGA AGG TGG ACA-3’ (antisense); GR, 5’-GGA GGT GAT TGA ACC CGA GG-3’ (sense), 5’-GCC TGG TAT CGC CTT TGC C-3’ (antisense); MR, 5’- GAA AAC AGA GGC TCA AGG TCA C-3’ (sense), 5’- CCT TGA GTT GTT GAG ATT TGC C-3’ (antisense); CRF1R, 5’-AGA TTG TAA AGC CTC TGG GTG TT-3’ (sense), 5’-GGT CTG ATA ATG CTT CCA GAT TTC T-3’ (antisense); CRF2R, 5’-CTC CTT GCT ACA CTG ACC CTT G-3’ (sense), 5’-GAG CCT CCA TTT CAT AGT TTT CC-3’ (antisense). The amplification efficiencies of β-actin, GR, MR, CRF1R, and CRF2R were E=100.3%, E=102.6%, E=101.4%, E=102.0%, and E=103.0%, respectively. Relative mRNA expression for each target was calculated by using the 2^–^^ΔΔCt^ method, ΔΔCt represents change relative to the control group, referenced to β-actin. 

***Preparation of brain tissue and immunohistochemistry for detection of GR, MR, CRF1R, and CRF2R***

The rats were anesthetized with 7% chloral hydrate, the heart and aortic arch were exposed, and infused with 200 ml of 37 °C saline through the ascending aorta. Subsequently, 100 ml of cold 4% paraformaldehyde (PFA) was infused at a rate of 50–60 drops per minute. After the infusion, the whole brain was removed rapidly, followed by 24–48 hr of post-fixation in 4% PFA at 4 °C. Rat brains were immersed in 30% sucrose in 0.1 M PBS until they sank to the bottom. And then the brain tissue blocks were trimmed according to the stereotaxic coordinates of the rat brain. The hippocampus and amygdala were located in 2.96–5.50 mm and 5.50–7.20 mm far away from the herringbone suture. Cut tissues were dehydrated and dipped into paraffin to make sections, 20 slides were reserved for each tissue. Following the immunohistochemical protocol, the endogenous peroxidase activity in the slides were blocked after dewaxing and hydration, the slides were then incubated with primary antibodies overnight (MR, rabbit polyclonal antibody, Lot: Ab64457; GR, rabbit polyclonal antibody, Lot: Ab3578; CRF1R, rabbit polyclonal antibody, Lot: Ab7786; CRF2R, rabbit polyclonal antibody, Lot: ab203585, all were purchased from Abcam company in Britain). After incubating with the secondary antibodies, DAB color rendering and hematoxylin staining were performed (Rabbit two-step detection kit, Lot: PV-9001; DAB color rendering kit, Lot: ZLI-9017), purchased from Beijing ZSGB-biotechnology co., LTD. Images in the hippocampus or amygdala were collected by the pathological image analysis system (400×), and positive cell count was conducted by Adobe Photoshop CS5.

***Statistical analysis***

All statistical analyses were performed using the SPSS 20.0 software package. The results were expressed as the mean±SEM. Statistical comparisons of the two groups were performed with unpaired Student’s t-test. Additional data were analyzed by one-way analysis of variance (ANOVA) followed by LSD’s multiple comparison tests. The level of significance was set for *P*<0.05 in all tests.

## Results

***Locomotor activities in OFT***

**Figure 3:**
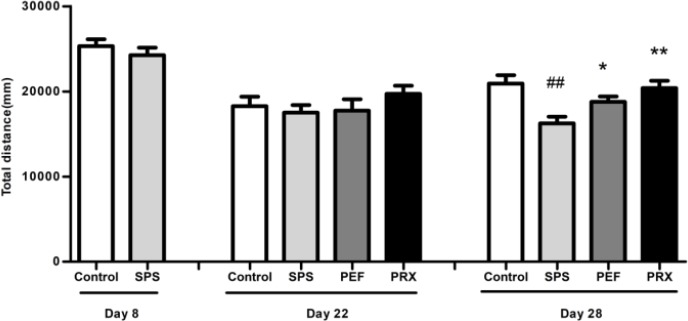
Effect of PEF on locomotor activity of rats. The behavior was presented by total distances (mm) in OFT. Values were expressed as mean±SEM (##*P*<0.01 vs control group, *P<0.05, **P<0.01 vs SPS group, n=10–12). PEF: paeoniflorin; OFT: open field test; SPS: single prolonged stress; PRX: paroxetine

As shown in Figure 3, no differences were found in total distance between groups on days 8 and 22 [*t*(18)=0.877, *P*=0.392; *F*(3,39)=0.794, *P*=0.505]. On day 29, total distance was significantly different between experimental groups [*F*(3,44)=6.559, *P*=0.001], and* post-hoc *test with LSD indicated that compared with that of the control group, total distance of SPS group significantly declined on day 29 (*P*<0.001), PEF and PRX rats significantly increased total distance vs the SPS group (*P*=0.035, *P*=0.001).

***Anxiety-like behavior in EPMT***

**Figure 4:**
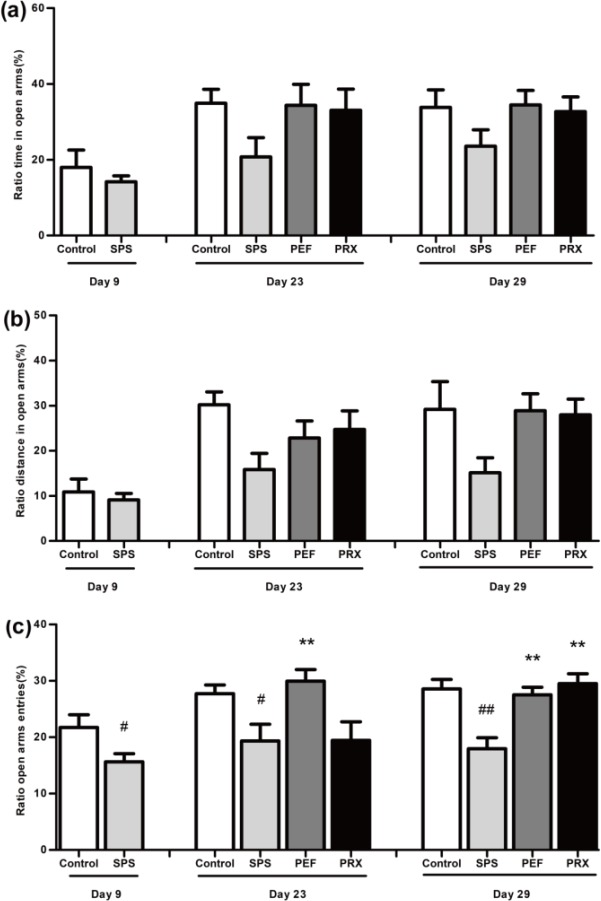
Effect of PEF on anxiety-like behaviors in EPMT. The behavior was presented by percentage of time (a), distance (b), and entries (c) in open arms. Values were expressed as mean±SEM (#*P*<0.05, ##*P*<0.01 vs control group, **P<0.01 vs SPS group, n=10–12)

PEF: paeoniflorin; EPMT: elevated plus-maze test; SPS: single prolonged stress; PRX: paroxetine

EPMT results were shown in Figure 4. Compared with the control group, the SPS group had significantly reduced percentage of entries in open arms on day 8 [*t*(17)=2.185, *P*=0.043; Figure 4c)]. On days 23 and 30, percentage of entries in open arms was significantly different between experimental groups [*F*(3,41)=4.775, *P*=0.006; *F*(3,44)=9.894, *P*<0.001; Figure 4c]. A* post-hoc *test with LSD indicated that the SPS group was lower than the control group in the percentage of entries in open arms on days 23 and 30 (*P*=0.019, *P*<0.001). PEF group was significantly higher than the SPS group in percentage of entries in open arms on days 23 and 30 (*P*=0.005, *P*<0.001). Compared with the SPS group, the PRX group significantly elevated the percentage of entries in open arms on day 30 (*P*<0.001) but not day 23 (*P*=0.978). There were no significant changes noted in the percentage of time and distance in open arms between groups on days 9, 23, and 30 [Day 9: corrected *t*(11.016)=0.773, *P*=0.456, Figure 4a; corrected *t*(13.178)=0.557, *P*=0.587, Figure 4b. Day 23: *F*(3,41)=2.760, *P*=0.054, Figure 4a; *F*(3,41)=1.806, *P*=0.161, Figure 4b. Day 30: *F*(3,44)=1.489, *P*=0.231, Figure 4a; *F*(3,44)=2.479, *P*=0.074, Figure 4b].

***ACTH levels in DST***

**Figure 5:**
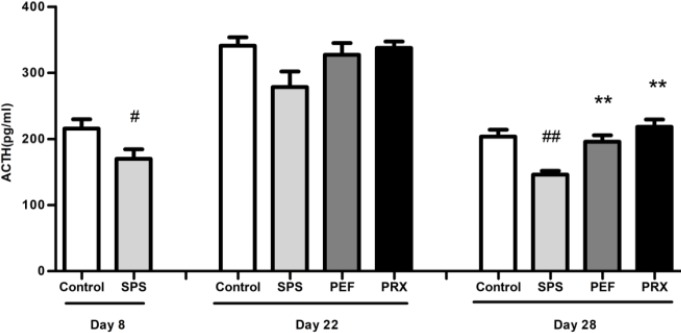
Effect of PEF on the plasma ACTH levels. Values were expressed as mean±SEM (#*P*<0.05, ##*P*<0.01 vs control group, **P<0.01 vs SPS group, n=8-12). PEF: paeoniflorin; ACTH: adrenocorticotropin hormone; EPMT: elevated plus-maze test; SPS: single prolonged stress; PRX: paroxetine

The results of DST were revealed in Figure 5. On day 8, the SPS group had significantly lower levels of plasma ACTH compared with the control group [*t*(16)=2.236, *P*=0.040]. There were significant changes noted in levels of plasma ACTH on day 29 [*F*(3,43)=10.589, *P*<0.001] but not on day 22 [*F*(3,29)=2.872, *P*=0.053]. A* post-hoc *LSD analysis showed significantly decreased plasma ACTH in the SPS group vs control group (*P*<0.001) and significantly increased plasma ACTH in PEF and PRX group vs SPS group (*P*=0.001; *P*<0.001).

***CORT and CRH detection***

**Figure 6:**
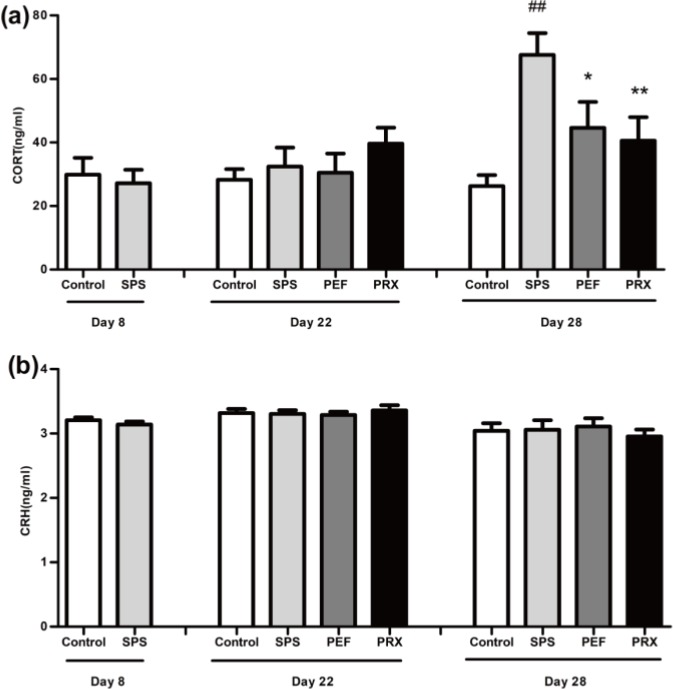
Effect of PEF on the levels of plasma CORT and CRH. The levels of plasma CORT were shown in a, and the levels of plasma CRH were shown in b. Values were expressed as mean±SEM (##*P*<0.01 vs control group, *P<0.05, **P<0.01 vs SPS group, n=10–12)

PEF: paeoniflorin; CORT: corticosterone; CRH: corticotropin releasing hormone; SPS: single prolonged stress; PRX: paroxetine

Results of CORT and CRH detection were illustrated in Figure 6. As shown in Figure 6a, there was significance in plasma CORT levels in the experimental group on day 29 [*F*(3,42)=6.586, *P*=0.001] but not on days 8 and 22 [*t*(22)=0.399, *P*=0.694; *F*(3,38)=0.812, *P*=0.495]. And* post-hoc *LSD analyses showed significantly increased plasma CORT levels in the SPS group vs control group (*P*<0.001). Plasma CORT levels in the PEF and PRX groups elevated significantly vs SPS group (*P*=0.021; *P*=0.006). As shown in Figure 6b, there were no significant difference in plasma CRH levels in any groups on days 8, 22, and 29[*t*(21)=1.009, *P*=0.324; *F*(3,40)=0.206, *P*=0.891; *F*(3,36)=0.257, *P*=0.856].

***RT-PCR for GR mRNA, MR mRNA, CRF1R mRNA, and CRF2R mRNA in the hippocampus and amygdala***

*RT-PCR results of the hippocampus*

**Figure 7:**
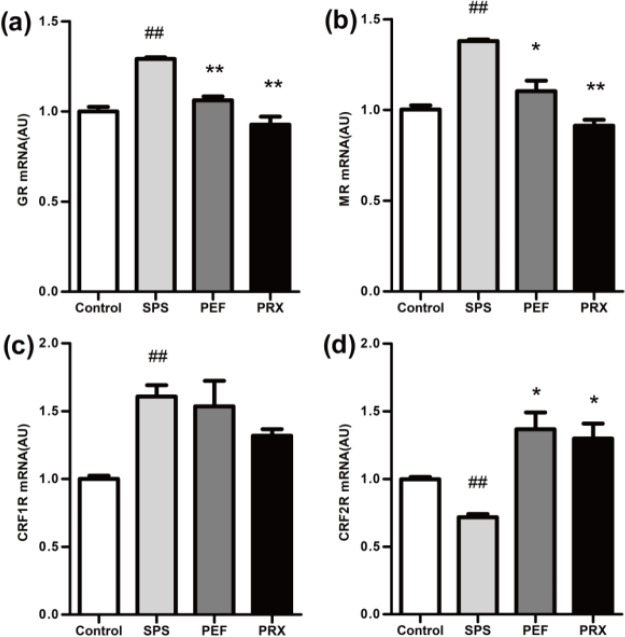
The mRNA expression of GR, MR, CRF1R, and CRF2R in the hippocampus was detected by RT-PCR. GR mRNA, MR mRNA, CRF1R mRNA, and CRF2R mRNA were shown in a–d. Values were expressed as mean±SEM (##*P*<0.01 vs control group, *P<0.05, **P<0.01 vs SPS group, n=6). GR: glucocorticoid receptor; MR: mineralocorticoid receptor; CRF1R: corticotropin releasing factor 1 recaptor; CRF2R: corticotropin releasing factor 2 recaptor; SPS: single prolonged stress; PEF: paeoniflorin; PRX: paroxetine

RT-PCR results of the hippocampus were shown in Figure 7. The expression of GR mRNA, MR mRNA, CRF1R mRNA, and CRF2R mRNA in the hippocampus were significantly different between groups [*F*(3,20)=31.465, *P*<0.001, Figure 7a; *F*(3,20)=32.520, *P*<0.001, Figure 7b; *F*(3,20)=6.594, *P*=0.003, Figure 7c; *F*(3,20)=12.490, *P*<0.001, Figure 7d]. A* post-hoc *test with Dunnett’s T3 indicated that GR mRNA, MR mRNA, and CRF1R mRNA significantly elevated and CRF2R mRNA significantly declined in the SPS group vs control group (*P*<0.001; *P*<0.001; *P*=0.002; *P*<0.001). In PFE and PRX group, GR mRNA and MR mRNA were significantly decreased and CRF2R mRNA was significantly increased in PEF and PRX groups vs SPS group (PEF: *P*<0.001; *P*=0.023; *P*=0.014. PRX: *P*<0.001;* P*<0.001; *P*=0.014). CRF1R mRNA had no statistical significance between the PEF and PRX vs SPS group (*P*=0.999; *P* = 0.079).

*RT-PCR results of the amygdala*

**Figure 8:**
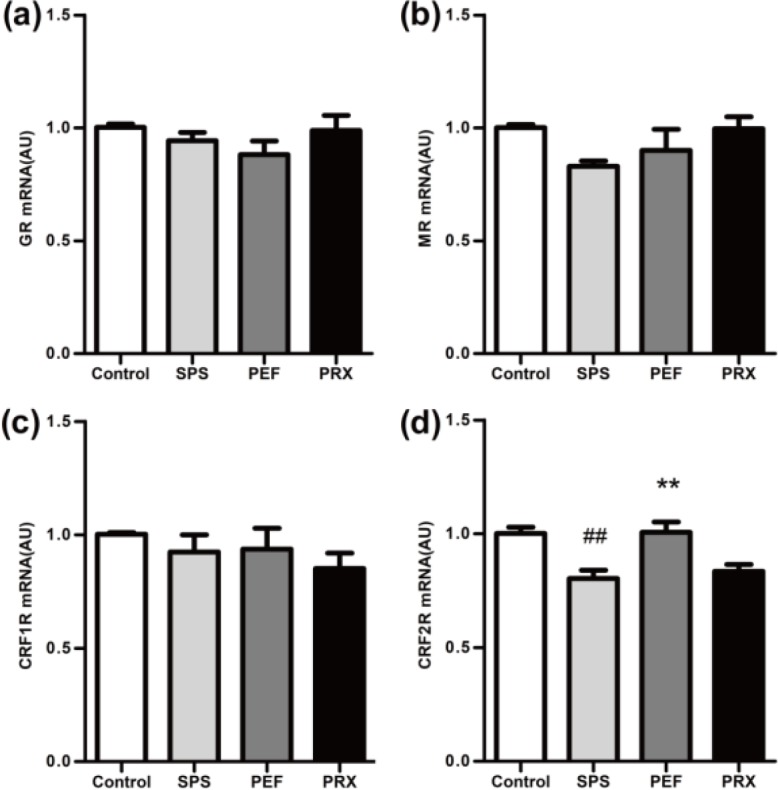
The mRNA expression of GR, MR, CRF1R, and CRF2R in the amygdala was detected by RT-PCR. GR mRNA, MR mRNA, CRF1R mRNA, and CRF2R mRNA were shown in a-d. Values were expressed as mean±SEM (##*P*<0.01 vs control group, **P<0.01 vs SPS group, n=6)

GR: glucocorticoid receptor; MR: mineralocorticoid receptor; CRF1R: corticotropin releasing factor 1 recaptor; CRF2R: corticotropin releasing factor 2 recaptor; SPS: single prolonged stress; PEF: paeoniflorin; PRX: paroxetine

RT-PCR results of the amygdala were shown in Figure 8. The expression of CRF2R mRNA of the amygdala showed statistical difference among groups [*F*(3,20)=9.088, *P*=0.001; Figure 8d]. The expression of GR mRNA, MR mRNA, and CRF1R mRNA had no significant changes [*F*(3,20)=1.233, *P*=0.324, Figure 8a; *F*(3,20)=2.183, *P*=0.122, Figure 8b; *F*(3,20)=0.550, *P*=0.654, Figure 8c]. A* post-hoc *test with LSD indicated that CRF2R mRNA was significantly decreased in the SPS group vs control group (*P*=0.001). PFE group CRF2R mRNA was significantly increased vs SPS group (*P*=0.001) and the PRX group had no statistical difference vs SPS group (*P*=0.538).

***Immunohistochemistry for GR, MR, CRF1R, and CRF2R in the hippocampus and amygdala***

*Immunohistochemistry results of the hippocampus*

**Figure 9:**
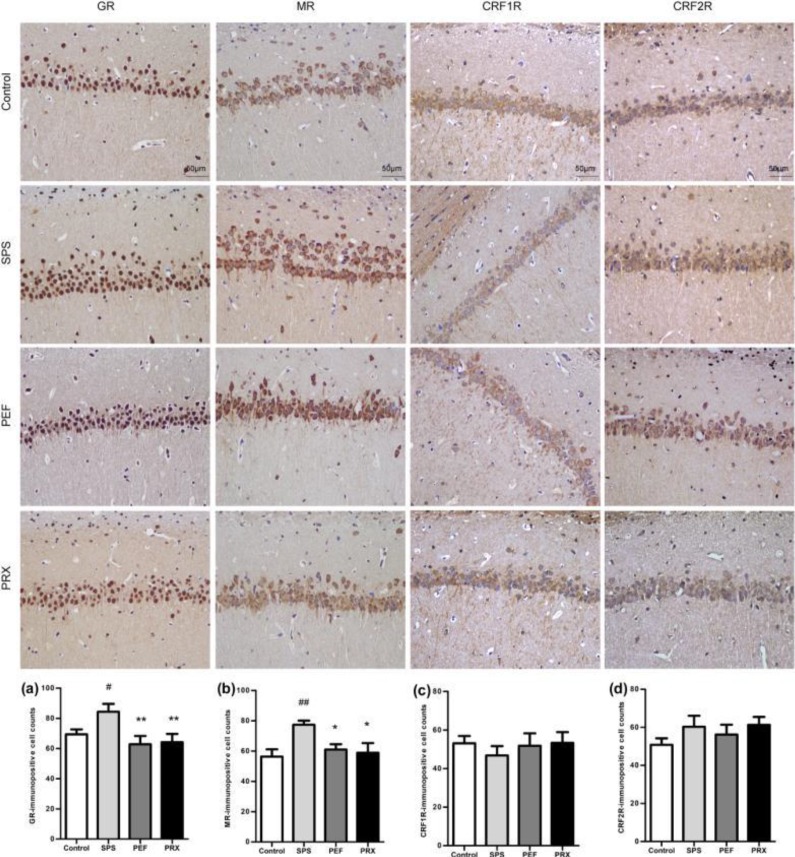
Expression of GR, MR, CRF1R, and CRF2R in the hippocampus of each group (magnification 400×) and results of analysis. Representative immunohistochemistry photomicrographs were shown on the top of this figure. The immunopositive cells' quantitative analysis of GR, MR, CRF1R, and CRF2R were shown in a-b. Values were expressed as mean±SEM (#*P*<0.05, ##*P*<0.01 vs control group, *P<0.05, **P<0.01 vs SPS group, n=6)

GR: glucocorticoid receptor; MR: mineralocorticoid receptor; CRF1R: corticotropin releasing factor 1 recaptor; CRF2R: corticotropin releasing factor 2 recaptor; SPS: single prolonged stress; PEF: paeoniflorin; PRX: paroxetine

Immunohistochemistry results of the hippocampus were shown in Figure 9. The immuno-positive cells were brown, and the count indicated that the number of immuno-positive cells of GR and MR had a statistically significant difference among groups [*F*(3,20)=3.948, *P*=0.023, Figure 9a; *F*(3,20)=3.622, *P*=0.031, Figure 9b]. The number of immuno-positive cells of CRF1R and CRF2R had no significant changes [*F*(3,20)=0.336, *P*=0.799, Figure 9c; *F*(3,20)=1.027, *P*=0.402, Figure 9d].* Post-hoc *with LSD comparisons further showed that there was significantly higher expression of hippocampus GR and MR immuno-positive cells in the SPS group than in the control group (*P*=0.045). GR and MR immuno-positive cells were significantly decreased in PEF and PRX group vs SPS group (PEF: *P*=0.006; *P*=0.031. PRX: *P*=0.009; *P*=0.017).

*Immunohistochemistry results of the amygdala*

**Figure 10:**
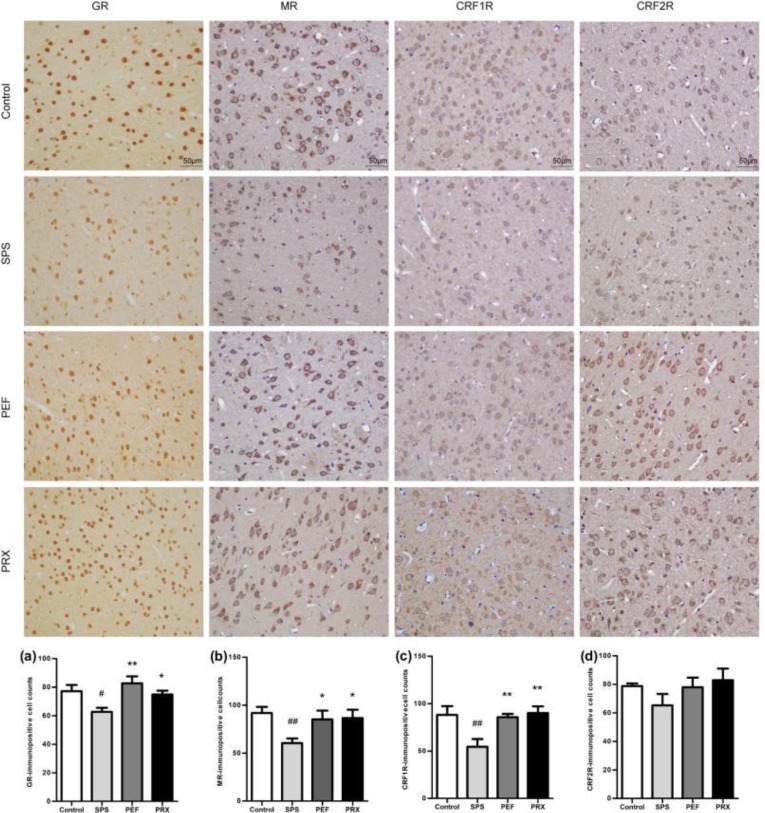
Expression of GR, MR, CRF1R, and CRF2R in the amygdala of each group (magnification 400×) and results of analysis. Representative immunohistochemistry photomicrographs were shown on the top of this figure. The immunopositive cells' quantitative analysis of GR, MR, CRF1R, and CRF2R were shown in a-b. Values were expressed as mean±SEM (#*P*<0.05, ##*P*<0.01 vs control group, *P<0.05, **P<0.01 vs SPS group, n=6)

GR: glucocorticoid receptor; MR: mineralocorticoid receptor; CRF1R: corticotropin releasing factor 1 recaptor; CRF2R: corticotropin releasing factor 2 recaptor; SPS: single prolonged stress; PEF: paeoniflorin; PRX: paroxetine

The immunohistochemistry results of the amygdala were shown in Figure 10. The count of brown immuno-positive cells indicated that there was significance in GR, MR, and CRF1R immuno-positive cells but not in CRF2R immuno-positive cells between experimental groups [*F*(3,20)=4.937, *P*=0.010, Figure 10a; *F*(3,20)=3.458, *P*=0.036, Figure 10b; *F*(3,20)=5.453, *P*=0.007, Figure 10c; *F*(3,20)=1.297, *P*=0.303; Figure 10d].* Post-hoc *with LSD comparisons further indicated that the expression of GR, MR, and CRF1R immuno-positive cells in the amygdala was significantly decreased in the SPS group vs control group (*P*=0.014; *P*=0.008; *P*=0.004). GR, MR, and CRF1R immuno-positive cells were significantly increased in PEF and PRX groups vs SPS group (PEF: *P*=0.001; *P*=0.031; *P*=0.006. PRX: *P*=0.035; *P*=0.024; *P*=0.002).

## Discussion

PTSD is a delayed and/or persistent mental disorder that develops following the experience of an unusually threatening or a catastrophic event. PTSD is classically characterized by anxiety, intrusive symptoms, irritability, enhanced conditioned fear memory and decreased learning memory. In this study, OFT and EPMT were used to evaluate behavioral changes of rats. The OFT is a good method to detect the locomotor activity of rats by utilizing their behavior of exploratory and wall-orientation in a new environment (24). EPMT examines the anxiety state of rats by taking advantage of the conflict between the exploration characteristics of animals to new environments and the fear of high hanging and open arms (25). Consistent with other studies (26), OFT results indicated that locomotor activities of the SPS group had not changed on days 8 and 22, but decreased significantly on day 29 after SPS. Anxiety levels of model rats in EPMT increased from day 9 to day 30 after SPS, which was in accordance with another report (27). It was found that after 14 days of continuous administration, PEF significantly attenuated the anxiety-like behaviors induced by SPS, and after 21 days of continuous treatment, PEF could significantly improve the locomotor activity. These results suggested that PEF has a good prospect in the application of anti-PTSD.

Changes in the HPA axis function have been a major concern in the study of PTSD. CORT is the terminal hormone secreted by the HPA axis and is often used to evaluate the basic function of the HPA axis. CORT presents a long-term dynamic change in PTSD patient, hence, in both clinical and rodent studies, different CORT levels in peripheral blood are obtained at different time points (28-30). In this study, we dynamically observed the changes of plasma CORT in each group from day 8 to day 29 after SPS. The results showed that CORT levels have no significant differences on days 8 and 22, but increased obviously on day 29, which was consistent with the literature reports (26, 29, 30). Elevated CORT levels are an important cause of anxiety. Besides being directly related to anxiety (31), it also may affect the processing of information and thus affect the behavioral response to specific stress. After 21 days of continuous treatment, PEF could obviously decrease plasma CORT levels, reduce the excitability of the HPA axis and adjust the HPA axis basic function to regulate the behavior abnormality of rats.

CRH is the initiate hormone secreted by the paraventricular nucleus (PVN) to activate the HPA axis and is another important index to evaluate the function of the HPA axis. The plasma CRH levels had no significant changes in model rats in this study, which was inconsistent with the changes in plasma CORT levels. It might be due to the distribution characteristics of CRH neurons. Besides the hypothalamus, CRH neurons are widely distributed in the central nervous system and multiple organs of the body (32, 33). The plasma CRH levels in peripheral blood have many sources, and the most important CRH for HPA axis regulation comes from small cell neurons in the PVN of the hypothalamus. Therefore, CRH and CORT in peripheral blood may show inconsistent changes.

The obvious enhanced HPA axis negative feedback is an important feature that differs from other mental diseases of PTSD (12, 34). Liberzon *et al.* used a stress-restress paradigm to compare plasma ACTH levels after 30 min restraint of rats injected with and without cortisol, and found that with the enhancement of HPA axis negative feedback, the levels of ACTH in rats decreased obviously (20). In this study, we used the same paradigm and found that the plasma ACTH levels of the SPS group significantly decreased on days 8 and 29 after SPS, indicating that the enhanced HPA axis negative feedback had occurred on days 8 and 29. As to the reason why did plasma CORT levels rise instead of falling when the negative feedback was enhanced, some studies suggest that enhanced negative feedback is a manifestation of the overall process of the HPA axis dysfunction, when the body gradually adapted to that dysfunction, the sensitivity of CORT to ACTH decreased, and then the secretion decreased (35). In addition, it has been reported that cortisol secretion in PTSD patients is characterized by a time series. Which increases first, then remains at a high level, then decreases, and finally continues at a low level (11, 29, 30). In this study, the model rats on day 29 might still be in the PTSD compensatory stage, which resulted in elevated plasma CORT levels. As the body gradually adapted to the HPA axis dysfunction, plasma CORT levels could decrease. Some studies have found that except for detecting time, gender may account for inconsistency of the cortisol levels in the PTSD patients (8). The cortisol level decrease is usually observed in studies of women with PTSD, while in studies of men with PTSD, cortisol levels often appear to be elevated or unchanged (8, 9, 36). The increase of CORT levels observed in this study may be related to the gender selection of rats. Considering the cyclical changes of sex hormones in females may affect the results, male rats were selected in this study (37). After 21 days of continuous treatment, PEF could obviously decrease plasma CORT levels and inhibit the enhanced negative feedback, which may also be an important aspect of PEF anti-PTSD.

The hippocampus and amygdala are high adjustment centers of the HPA axis, which plays an important role in the regulation of the basic function and negative feedback function of the HPA axis. The activation of the hippocampus mainly causes the HPA axis negative feedback (18), which reduces the CORT release, while the activation of the amygdala mainly promotes the excitability of the HPA axis and the release of CORT (19). This functional difference may be related to the expression of GR and MR receptors in the hippocampus and amygdala.

Consistent with another report (38), the PTSD model rats had a significantly increased expression of GR in the hippocampus in this study. The HPA axis negative feedback regulation is mainly mediated after the combination of CORT and GR in the hippocampus (39). In the SPS group, the increased GR expression of the hippocampus may be an important reason for the enhanced negative feedback. On the other hand, the expression of MR in the hippocampus is mainly related to maintaining the excitability of the hippocampus, the basic function of the HPA axis, and the sensitivity to pressure (40). The levels of MR expression in the hippocampus determine the basic levels of CORT, and the expression of hippocampus MR increased significantly in this study, which may be one of the reasons for the increased excitability of the HPA axis and the increase of plasma CORT levels on day 29. It has been found that the increased expression of GR in the hippocampus is directly related to the retention of fear memory (41), which leads to fear, anxiety, and other manifestations of PTSD. The activation of MR can regulate memory, behavior, anxiety, and fear (39, 42). These studies suggested that the increased expression of GR and MR in the hippocampus may also be connected with the anxiety-like behavior and decreased autonomic activity in the model rats.

Currently, it is generally believed that both GR and MR in the amygdala promote the secretion of CORT (43). Our study found that PTSD model rats had decreased expressions of GR and MR in the amygdala, which was thought to be associated with reduced basic CORT levels in general studies (43). Novelly, the CORT levels were elevated in this study, probably due to co-regulation by MR in the hippocampus and GR and MR in the amygdala (44). On day 29 the model rats may be still in the compensatory stage of HPA axis dynamic changes (45). At this stage, elevated MR in the hippocampus might have a dominant effect on CORT, leading to increased CORT levels. 

Decreased expressions of GR and MR in the amygdala are associated with potential fear learning and play an important regulatory role in fear emotional response (44), which might be one of the reasons for behavioral abnormalities in model rats. After 21 days of continuous treatment, PEF could significantly reduce the expression of GR and MR in the hippocampus and increased the expression of GR and MR in the amygdala, which might be why PEF inhibited the enhanced HPA axis negative feedback, reduced the plasma CORT levels, and alleviated behavioral abnormalities.

CRF1R and CRF2R are two receptors of CRH. In this study, it was shown that CRF1R mRNA in the SPS group was significantly increased in the hippocampus while CRF2R mRNA was significantly decreased in the hippocampus and amygdala. Especially, only the number of CRF1R immune positive cells in the amygdala decreased significantly. The results suggested that the protein expressions of CRF1R and CRF2R were regulated by various factors. The expression of CRF1R and CRF2R in the hippocampus was less affected by SPS, while the expression of CRF1R in the amygdala was sensitive to SPS. Activation of CRF1R is mainly associated with increased anxiety levels, but its function varies in different brain regions (33, 46, 47). In the amygdala, the effect caused by CRF1R activation is related to the stress response of the body (48). Meanwhile, CRF1R antagonists have been found to increase conditioned fear in both healthy persons and rodents (49, 50), its mechanism may be in connection with the direct projection to globus pallidus lateralis by CRH neurons in the amygdala (51). With the decreased expression of CRF1R in the amygdala, the globus pallidus lateralis projection by CRH neurons in the amygdala decreased, which increased the response to stress and reduced the anxiety levels. In this study, it was found that the decreased expression of CRF1R in the amygdala might be related to the anxiety-like behavior of the model rats. PEF could improve the anxiety-like behavior in rats, which may be caused by significantly increased expression of CRF1R in the amygdala.

## Conclusion

Taken together, PEF could reduce anxiety-like behavior, improve the autonomic activity and exert the anti-PTSD effect. Its mechanism may be related to regulating the expressions of GR, MR, and CRF1R in the hippocampus and amygdala, and further inhibiting the enhanced HPA axis negative feedback function.

## Data Availability

The data in this article was used to support the findings of this study.
